# Acetylation of the histone H3 tail domain regulates base excision repair on higher-order chromatin structures

**DOI:** 10.1038/s41598-019-52340-0

**Published:** 2019-11-04

**Authors:** Deb Ranjan Banerjee, Charles E. Deckard, Yu Zeng, Jonathan T. Sczepanski

**Affiliations:** 1 Department of Chemistry, Texas A&M University, College Station, Texas 77843 United States; 2 Present Address: Department of Chemistry, National Institute of Technology, Durgapur, West Bengal India; 3 Present Address: Department of Microbial Pathogenesis and Immunology, Texas A&M University, College Station, Texas 77843 United States

**Keywords:** Base excision repair, Nucleic acids

## Abstract

Despite recent evidence suggesting that histone lysine acetylation contributes to base excision repair (BER) in cells, their exact mechanistic role remains unclear. In order to examine the influence of histone acetylation on the initial steps of BER, we assembled nucleosome arrays consisting of homogeneously acetylated histone H3 (H3K18 and H3K27) and measured the repair of a site-specifically positioned 2′-deoxyuridine (dU) residue by uracil DNA glycosylase (UDG) and apurinic/apyrimidinic endonuclease 1 (APE1). We find that H3K18ac and H3K27ac differentially influence the combined activities of UDG/APE1 on compact chromatin, suggesting that acetylated lysine residues on the H3 tail domain play distinct roles in regulating the initial steps of BER. In addition, we show that the effects of H3 tail domain acetylation on UDG/APE1 activity are at the nucleosome level and do not influence higher-order chromatin folding. Overall, these results establish a novel regulatory role for histone H3 acetylation during the initiation of BER on chromatin.

## Introduction

Cellular DNA is constantly exposed to genotoxic agents arising from both exogenous and endogenous sources. If left unrepaired, the resulting DNA lesions can lead to genetic instability and alterations that are at the heart of various diseases, including cancer, cardiovascular diseases, and neurodegenerative diseases, as well as aging^1–5^. The front-line defense against damaged nucleobases is the base excision repair (BER) pathway^6^. BER is initiated by DNA glycosylases that are responsible for recognizing and excising specific nucleobase lesions via cleavage of the glycosidic bond^7^. For example, 2′-deoxyuridine (dU), which can result from the deamination of cytosine^8^, is recognized and excised from DNA by uracil DNA glycosylase (UDG). Removal of the damaged base results in formation of an abasic site, which is subsequently cleaved by apurinic/apyrimidinic endonuclease 1 (APE1) in order to generate a nicked substrate for further downstream processing^6^.

Like all genomic processes, BER must take place within the structural constraints of chromatin. In eukaryotic cells, the basic unit of chromatin is the nucleosome, which consists of ∼147 base pairs (bp) of DNA wrapped nearly two times around a protein core comprising two copies of each histone H2A, H2B, H3, and H4. In general, nucleosomes are repressive structures that restrict DNA-binding factors from accessing the underlying DNA^9,10^. Indeed, nearly every step in the BER process is inhibited to varying degrees by the packaging of DNA into nucleosomes, and the extent of inhibition depends largely on the rotational setting of the lesion in relation to the histone octamer surface^11–15^. In addition, individual nucleosomes are connected by stretches of linker DNA to form chromosome-sized nucleosome arrays that fold and condense into more compact higher-order structures that further impact DNA accessibility and BER^16–18^. For example, the combined catalytic activities of UDG and APE1 are inhibited by as much as 20-fold upon folding of nucleosome arrays into compact 30 nm chromatin fibers^19^.

Given the impact of chromatin structure on BER, it is not surprising that histone posttranslational modifications (PTMs) have been implicated in the repair process^20–23^. In particular, histone acetylation has been shown to directly impact nucleosome stability, mobility, and unwrapping^24–26^, as well as higher-order chromatin folding^27,28^, supporting a model in which histone acetylation directly regulates BER by controlling DNA accessibility. Indeed, emerging evidence supports a role for site-specific histone acetylation in cellular BER^29,30^. However, due to challenges associated with manipulating the chemical structure of chromatin in cells, functional relationships between histone acetylation and BER remain mostly unexplored. A few pioneering studies have investigated the influence of histone acetylation on BER using homogeneously acetylated mononucleosomes as model substrates^30,31^. Uncoupled from the more complex cellular environment, reconstituted mononucleosomes enable quantitative interrogation of the BER pathway in the context of specific PTMs. Although these studies have provided novel insight into how acetylation potentially regulates BER *in vivo*, mononucleosomes fall short of being an ideal chromatin model. In particular, they cannot be used to assess the impact of histone acetylation (or other PTMs) on inter-nucleosomal interactions and higher-order chromatin folding, which are likely key factors that regulate BER at the chromatin level^19,32^.

In the present study, we assembled novel nucleosome arrays consisting of both site-specifically damaged DNA and homogeneously acetylated histones in order to investigate, for the first time, the impact of histone acetylation on BER within higher-order chromatin structures. Specifically, we reconstituted 12-mer nucleosome arrays consisting of one of two homogeneously acetylated H3 histones (H3K18ac and H3K27ac) and measured the repair of a site-specifically positioned dU residue by the combined activities of UDG and APE1. By comparing acetylated chromatin substrates, including mononucleosomes, to their unmodified counterparts, we find that H3K18ac and H3K27ac modulate the activities of UDG and APE1 in a context-dependent manner, indicating that acetylation of histone H3 has a site-specific effect on BER. Given that H3K18 and H3K27 are targets of the acetyltransferase CBP/p300, this work potentially establishes a novel regulatory role for CBP/p300 in the BER pathway.

## Results and Discussion

### Assembly of chemically defined nucleosome arrays

**Figure 1:**
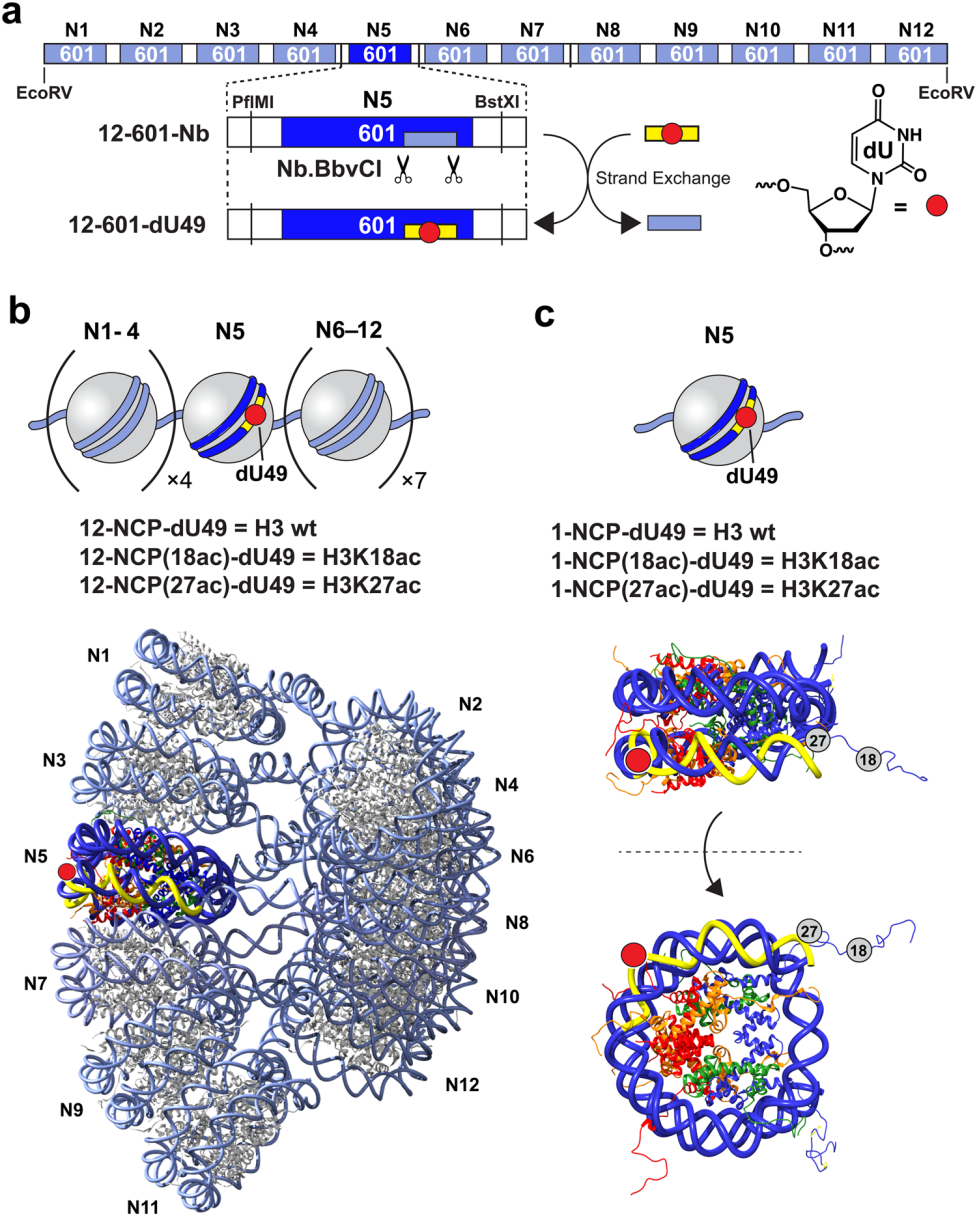
Chromatin substrates containing site-specifically modified DNA and histone H3. (**a**) Schematic illustration of the 12 × 601 DNA template used in this study. The exchangeable DNA fragment within nucleosome 5 (N5) (**12-601-Nb**, light blue box) is indicated. (**b**) The nucleosome arrays prepared in this study (top) and the cryo-electron microscopy structural model of the 30 nm chromatin fiber (12 × 601, EMD-2600) showing the locations of dU49, as well as the corresponding Nb.BbvCI (yellow) excisable DNA region (bottom). The nucleosome surface (PDB ID: 1ZBB) was fitted to the electron density map using Chimera^79^. (**c**) The mononucleosomes prepared in this study (top) and corresponding x-ray structure **(**PDB ID: 1ZBB) showing the position of dU49 and acetylated lysine residues (H3K18ac and H3K27ac). See Supporting Information for DNA sequence information.

**Figure 2:**
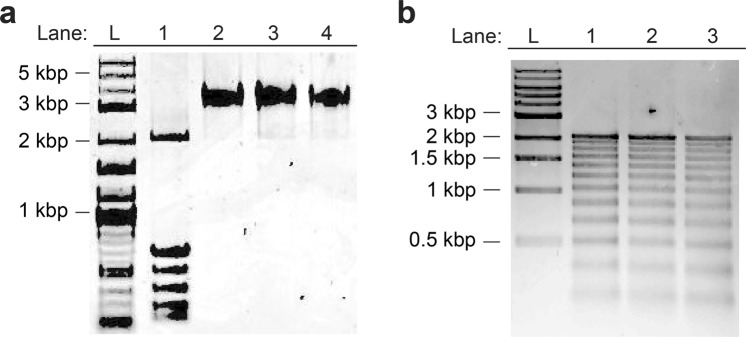
Characterization of modified 12-mer nucleosome arrays. (**a**) Purified nucleosome arrays used in this study. Lane 1, **12-601-dU49** prior to reconstitution (for reference); lane 2, **12-NCP-dU49**; lane 3, **12-NCP(27ac)-dU49**; lane 4, **12-NCP(18ac)-dU49**. (**b**) Partial MNase digestion of nucleosome arrays used in this study. Lane 1, **12-NCP-dU49**; lane 2, **12-NCP(27ac)-dU49**; lane 3, **12-NCP(18ac)-dU49**. Molecular size markers are indicated in both panels (L). Uncropped images are presented in Fig. S9.

The nucleosome arrays described in this work utilize a DNA template composed of 12 copies of the “ Widom 601” nucleosome position sequence^33^, each of which is separated by 30 bp of linker DNA (Figs 1a and S1a). This array architecture has been shown to reconstruct the native chromatin environment and the spatial relationship between neighboring nucleosomes^34,35^, and has been used extensively to investigate the regulation of chromatin structure by histone PTMs^28,36–38^. Furthermore, while the 601 DNA sequence has a high affinity for the histone octamer, mononucleosomes and arrays assembled using 601 DNA remain highly dynamic and undergo spontaneous conformational fluctuations that increase DNA accessibility (i.e. site exposure) on a biologically relevant timescale^39–41^. We designed the 12 × 601 template (**12-601-Nb**) to contain two proximal nicking endonuclease recognition sites (Nb.BbvCI) within the nucleosome-bound region of the fifth 601 repeat (N5), which enabled site-specific incorporation of single dU residue using our previously reported “plug-and-play” approach (see “Methods” section and Fig. S1)^19^. The minor sequence variations resulting from the inclusion of these restriction sites do not significantly alter the rotational positioning of the DNA relative to the native 601 sequence^19^. Treatment of **12-601-Nb** with Nb.BbvCI resulted in formation of a short gap upon heat denaturation, which was subsequently filled in with a DNA oligonucleotide carrying the desired dU residue (Table S1). The remaining nicks were then resealed by T4 DNA ligase, resulting in generation of the fully intact DNA template (**12-601-dU49**). Complete incorporation (>95%) of the dU-modified oligonucleotide was confirmed using an electrophoretic mobility retardation assay (Fig. S1b). The final position of the dU residue (49 nucleotides from the N5 dyad; dU49) was such that upon reconstitution of DNA template into chromatin, the lesion would be facing outward relative to the underlying nucleosome surface (Fig. 2b). UDG/APE1-mediated digestion of dU at this position is highly sensitive to magnesium-induced chromatin compaction, suggesting that this position is ideal for examining the impact of histone PTMs on the initial steps of BER^19^.

The dU containing DNA template (**12-601-dU49**) was reconstituted into 12-mer nucleosome arrays using recombinant human histone octamers containing either wild-type (WT) H3 or homogeneously acetylated H3 (H3K18ac or H3K27ac)^42–44^. The acetylated H3 proteins were produced via unnatural amino acid mutagenesis using an amber suppression-based approach (Fig. S3)^45^. Optimal reconstitution conditions were determined by histone octamer titration, and fully saturated nucleosome arrays were purified via selective magnesium-induced precipitation (Fig. S4a)^43,46,47^. Native gel electrophoresis of the reconstituted products revealed, in all cases, a single band consistent with a homogeneous population of nucleosome arrays (Fig. 2a). Partial digestion with micrococcal nuclease (MNase) confirmed the presence of 12 positioned nucleosomes (Fig. 2b), and restriction enzyme digests of the reconstituted arrays demonstrated full nucleosome occupancy (Fig. S4b). Together, these data suggest that formation of nucleosome arrays is not affected by the presence of a single dU residue and acetylated histone H3, which is in agreement with several previous reports^11,19,30,32^.

### Uracil removal by UDG and APE1 is dependent on site-specific histone acetylation

We investigated the impact of H3 acetylation (H3K18ac and H3K27ac) on the combined activities of UDG/APE1. We chose these lysine residues because they are preferred targets for the acetyltransferase p300 and closely related CBP (CBP/p300)^48,49^. However, additional sites on the histone H3 tail (e.g. K9, K14, and K23) can also be acetylated by CBP/p300. CBP/p300 has been shown to interact with and acetylate a number of key enzymes in the BER pathway, including several DNA glycosylases, APE1, and polymerase β (pol β)^50–53^. Thus, it is possible that these BER proteins could facilitate the recruitment of CBP/p300 to sites of DNA damage to acetylate histones and potential stimulate BER^54^. However, this has yet to be experimentally demonstrated.

We examined UDG/APE1-mediate repair of dU under both low (0.2 mM) and high (2.0 mM) Mg^2+^ concentration. At 0.2 mM Mg^2+^, 12-mer arrays take on an extended “beads-on-a-string” structure representing an accessible chromatin state. However, at 2 mM Mg^2+^, these same arrays fold into compact “30 nm” fibers^55,56^. Magnesium has been shown to impact the activities of UDG enzymes and is required for APE1 catalysis^6,57–59^. Magnesium also influences nucleosome dynamics and stability^60,61^. Therefore, the rate of BER on each substrate type (naked DNA, mononucleosomes, and arrays) was measured at both Mg^2+^ concentration and only datasets obtained at the same Mg^2+^ concentration (i.e. chromatin structural state) were compared to each other.

**Figure 3:**
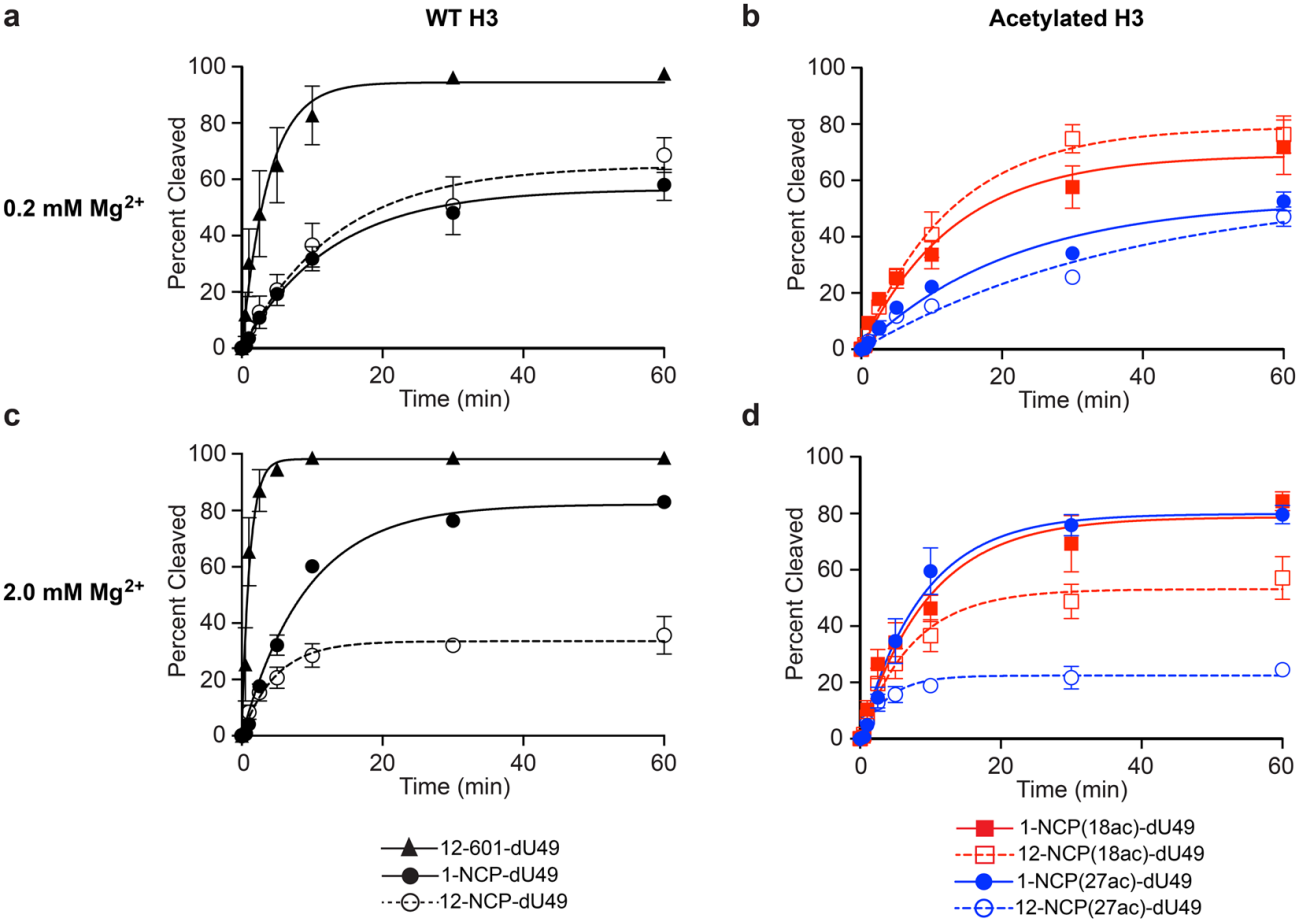
BER of damaged DNA within various chromatin environments. The indicated substrates (10 nM; Fig. 1) were incubated with UDG and APE1 (1 nM each) in the presence of either 0.2 mM Mg^2+^ (**a,b**) or 2.0 mM Mg^2+^ (**c,d**). Error bars represent standard deviation from at least three independent experiments. See Table 1 for relevant statistics.

We first examined the ability of UDG/APE1 to digest the various chromatin substrates (Fig. 1b) under low magnesium conditions (0.2 mM Mg^2+^), where nucleosome arrays are mostly in an extended state^55,56^. Both naked DNA (**12-601-Nb**) and nucleosome arrays (10 nM, Fig. 1b) were treated with UDG and APE1 (1 nM each), and cleavage of the dU-containing strand was measured over time (Figs 3a,b and S6a). A similar set of digestion reactions was also carried out on either unmodified or acetylated (H3K18ac and H3K27ac) mononucleosome substrates containing an identically positioned dU lesion (Figs 1c and S2, S6b). We note that the reaction conditions used here have been used extensively to study the impact of nucleosomes on UDG/APE-mediated BER^11,12,62,63^. In agreement with our previous study^19^, UDG/APE1-mediated digestion of dU49 was significantly inhibited on both mononucleosomes and nucleosome arrays relative to free DNA (Fig. 3a and Table 1). Importantly, the activity of UDG/APE1 on the chromatin substrates was dependent on histone H3 acetylation. While H3K18ac had no effect on digestion of dU49 by UDG/APE1, H3K27ac resulted in significant inhibition of UDG/APE1 activity relative to WT histone H3 (Fig. 3b and Table 1). This trend was similar for both mononucleosomes and nucleosome arrays, suggesting that the influence of H3K27ac on UDG/APE1 activity is likely the result of intra-nucleosomal interactions rather than interactions between neighboring nucleosomes. This is consistent with previous observations that acetylation of the H3 tail domain primarily regulates DNA accessibility at the nucleosome level^27^. This is also in agreement with our previous observation that accessibility of dU49 in extended arrays is almost exclusively dictated by the underlying histone octamer^19^. These different outcomes were somewhat surprising given the close proximity of these two lysine residues to each other on the histone H3 tail (Fig. 1c). Furthermore, previous work has shown that acetylation of lysine residues near the N-terminus of the H3 tail domain (e.g. H3K14 and H3K18) does not induce significant changes in nucleosome dynamics (i.e. DNA unwrapping)^30,64^. Nevertheless, acetylation has been shown to alter H3 tail dynamics and tail-DNA contacts^65,66^. Therefore, it is possible that H3K27ac, but not H3K18, results in the repositioning of the H3 tail domain in such a way that accessibility of dU49 to UDG/APE1 is reduced. Further experiments are needed to verify this model.

**Table 1:** Initial rates of cleavage and extent digestion of dU49 by UDG/APE1.

[Mg2+] (mM)	Substrate	*k* (% cleaved per minute)
0.2 mM	12-601-dU	24.8 ± 2.6
	1-NCP-dU	4.6 ± 0.3^a^
	12-NCP-dU	4.7 ± 0.8^a^
	1-NCP(18ac)-dU	5.6 ± 0.8^a^
	12-NCP(18ac)-dU	6.4 ± 0.4^a^
	1-NCP(27ac)-dU	2.4 ± 0.5^a,b^
	12-NCP(27ac)-dU	1.5 ± 0.6^a,b^
2.0 mM	12-601-dU	86.2 ± 9.8
	1-NCP-dU	8.2 ± 0.8^a^
	12-NCP-dU	6.8 ± 0.8^a^
	1-NCP(18ac)-dU	8.2 ± 1.4^a^
	12-NCP(18ac)-dU	6.9 ± 1.0^a^
	1-NCP(27ac)-dU	9.1 ± 1.0^a^
	12-NCP(27ac)-dU	6.0 ± 0.9^a^

Initial rates were calculated by fitting the digestion data in Fig. 3 to a single phase exponential and multiplying each rate constant by the *Y*_max_ as previously described^12,63^. ^a^P < 0.05 compared to naked DNA. ^b^P < 0.05 compared to non-acetylated substrate.

**Figure 4:**
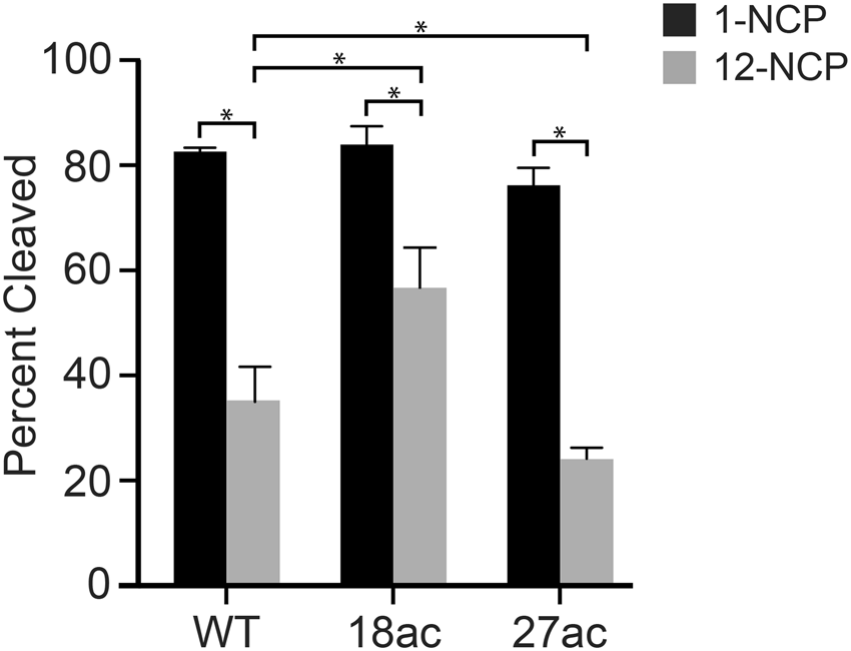
Acetylation of histone H3 differentially regulates UDG/APE1-mediated removal of dU49 on compact chromatin. The indicated substrates (10 nM; Fig. 1) were incubated with UDG and APE1 (1 nM each) in the presence of 2.0 mM Mg^2+^ for 60 minutes. Error bars represent standard deviation from at least three independent experiments (*P < 0.05).

Next, we repeated the UDG/APE1 digestion experiment described above in the presence of 2 mM Mg^2+^, which induces compaction of the extended array into a “30 nm” chromatin fiber^55,56^. Consistent with our previous observations^19^, increasing the Mg^2+^ concentration from 0.2 to 2.0 mM stimulated UDG/APE1 activity on both free DNA and mononucleosomes (Table 1). Under these conditions, we found that acetylation of either H3K18 or H3K27 did not significantly affect the rate of UDG/APE1-mediated cleavage of dU49 on mononucleosomes (Fig. 3c,d and Table 1). The extent of digestion (∼80%) was also independent of acetylation (Fig. 4). Digestion of compact arrays by UDG/APE1 resulted in the formation of substantially less incised product overall compared to corresponding mononucleosome substrates (regardless of the acetylation state), which is consistent with our previous observation that folding of chromatin into a 30 nm fibers is a major obstacle for BER (Fig. 4)^19^. Importantly, the amount of dU49 digested by UDG/APE1 after 60 minutes on the compact arrays was highly dependent on the acetylation state of histone H3. Acetylation of H3K18 resulted in increased product formation relative to the unmodified array, whereas acetylation of H3K27 had the opposite effect (Figs 3c,d and 4). Notably, product formation was inhibited by only ∼30% on H3K18ac containing arrays (57.1 ± 7.6% cleavage) relative to mononucleosomes (83.0 ± 0.7% cleavage) after 60 minutes, whereas ∼70% inhibition of product formation was observed on H3K27ac arrays (24.5 ± 2.1% cleavage) (Fig. 4). Interestingly, despite differences in the amount of product formed, we found that the initial rate of dU49 incision was similar for each of the array substrates (Table 1). A possible explanation for this observation is the existence of at least two distinct substrate populations within each compact array: one in which dU49 is directly accessible to UDG/APE1 and one where it is not, which is consistent with the dynamic nature of nucleosome arrays^39,67^. Accordingly, our data suggests that acetylation of histone H3 influences the distribution of these states in a site-specific manner, with acetylation of H3K18 shifting the population towards a more accessible state. However, based on our steady-state analysis, we cannot rule out that product inhibition plays a role.

It was previously reported that acetylation of the histone H3 tail domain (H3K14) does not affect the combined activities of UDG/APE1 on damaged mononucleosome substrates^30^. These experiments were carried out under “high Mg^2+^” conditions (5 mM), and are in general agreement with our mononucleosome data presented in Fig. 3. However, by expanding these studies to include oligonucleosome arrays, which more accurately represent chromatin *in vivo*, the above results demonstrate that histone acetylation can have a dramatic affect on the initial steps of BER on compact chromatin. To the best of our knowledge, this is the first direct experimental evidence demonstrating that histone acetylation is capable of modulating UDG/APE1-mediated excision of dU from chromatin.

### Acetylation of the histone H3 tail domain regulates BER at the nucleosome level within compact chromatin

**Figure 5:**
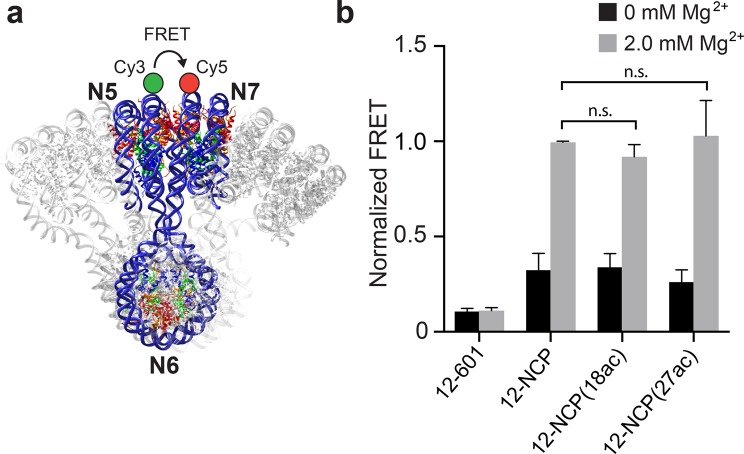
H3 acetylation does not impact higher-order chromatin structure. (**a**) Locations of the donor (Cy3) and acceptor (Cy5) for the FRET assay. (**b**) FRET analysis of nucleosome array compaction. Arrays were reconstituted using either WT histone H3 (12-NCP), H3K18ac (12-NCP(18ac)), or H3K27ac (12-NCP(27ac)). All fluorescent intensities were normalized to WT arrays (12-NCP) at 2.0 mM Mg^2+^. n.s. = Not significant.

In our previous study, we showed that digestion of dU49 on nucleosome arrays by UDG and APE1 was highly dependent on the structural state of the array^19^. In order to determine if structural differences exist between unmodified and acetylated arrays that may explain the observed variation in UDG/APE1 activity, we utilized a previously reported fluorescence resonance energy transfer (FRET)-based assay that measures the distance between nucleosome 5 (N5) and nucleosome 7 (N7) within the arrays (Figs 5a and S7)^39,68^. Mg^2+^-induced compaction (2.0 mM) of both WT and H3 acetylated arrays resulted in a dramatic increase in the FRET signal (>10-fold) compared to naked DNA (Fig. 5b), consistent with the formation of compact 30 nm chromatin fibers. Importantly, no significant difference in the extent of compaction (i.e. distance between nucleosomes) was observed for acetylated arrays relative to unmodified arrays, indicating that the higher-order structure was not perturbed by histone H3 acetylation.

**Figure 6:**
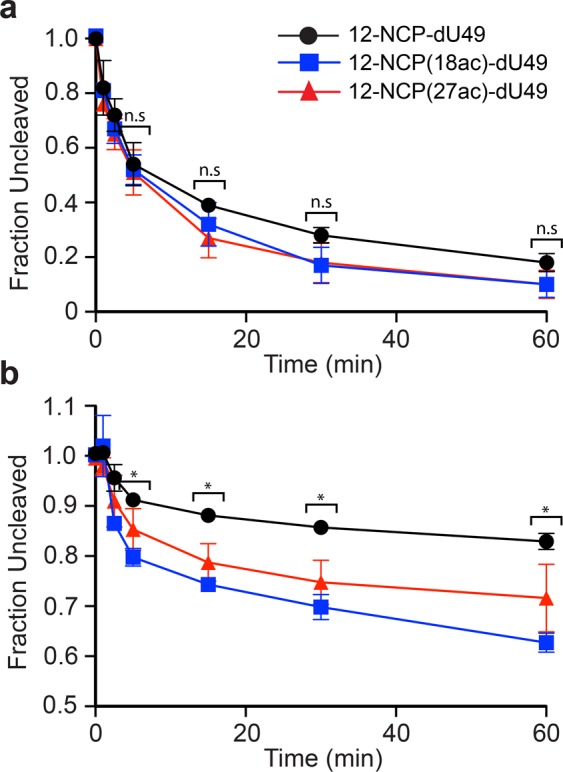
Restriction enzyme accessibility assay on compact chromatin arrays. The indicated substrate was incubated with either BstXI (**a**) or BbvCI (**b**) under high magnesium conditions (2.0 mM Mg^2+^). Error bars represent standard deviation from at least three independent experiments (P < 0.05 compared to non-acetylated substrate).

Next, we measured restriction enzyme (RE) accessibility within compact arrays (2 mM Mg^2+^) containing either WT or acetylated histone H3 (Fig. 6). Previous studies have shown that RE digestion serves as a sensitive assay to interrogate the influence of histone PTMs on chromatin structure and DNA accessibility^27,69^. We first assessed the rate of cleavage of the BstXI restriction site, which is positioned within the linker DNA between the fifth (N5) and sixth (N6) nucleosomes in the array (Fig. 1a). We found no significant difference in the rate of cleavage by BstXI between these arrays, indicating that H3K18ac and H3K27ac do not cause significant changes in the overall structure of compact arrays (Figs 6a and S8a). This is in agreement with previous work showing that installation of up to six acetylation mimics (i.e. K → Q substitutions) in the H3 tail domain does not increase accessibility within nucleosome arrays^27,69^. We then investigated the cleavage of two BbvCI restriction sites, which are positioned on either side of dU49 within the nucleosome (N5) core (BbvCI and Nb.BbvCI share the same recognition sequence) (Fig. 1a). In contrast to BstXI, cleavage by BbvCI within the nucleosome was significantly greater on both acetylated arrays compared to the WT array, indicating that acetylation of the H3 tail domain increases DNA accessibility near dU49 (Figs 6b and S8b).

Together, these data suggest that the observed effects of acetylation on UDG/APE1-mediate BER in compact chromatin is not due to changes in the higher-order structure, but rather acetylation of the H3 tail domain likely influences nucleosome-level accessibility of the DNA near dU49 in a lysine-dependent manner. Interestingly, these results show that RE accessibility and UDG/APE1 activity are not always correlated, further demonstrating the complex nature of BER on chromatin.

## Conclusions

Despite the need for BER to occur throughout the genome, it is now clear that nucleosome organization and chromatin structure appear to restrict this pathway^70–73^. Indeed, several studies have observed an inverse correlation between BER and chromatin compaction in living cells^72,73^. For example, the mammalian 8-oxoguanine DNA glycosylase OGG1 is excluded from heterochromatin regions upon induction of oxidative DNA damage^72^. Furthermore, a recent genome-wide mapping study revealed that dU is enriched within the highly compact environment of human centromeres^73^. Our results obtained using nucleosome arrays are consistent with these observations in cells (Fig. 3), and show that inter-nucleosomal interactions and histone tail domains play a significant role in regulating UDG/APE1-mediated repair of dU within compact chromatin.

In order to investigate how histone acetylation impacts the repair of dU within chromatin, we assembled nucleosome arrays containing both site-specifically damaged DNA and homogeneously acetylated histones. Our results demonstrate that acetylation at a particular site does not have a general effect on UDG/APE1 activity, but rather each site must be considered individually. However, because we only examined a single positioned dU residue, we cannot rule out that H3K18ac and H3K27ac may have a different effect on the removal of dU at other positions, for example within the linker DNA. Similarly, acetylation of other lysine residues, including those on histone H4, may result in alternative outcomes. Nevertheless, the opposing roles of histone H3 acetylation during BER observed here highlights the complex nature of this relationship and suggests that regulation of BER in chromatin likely requires precise-tuning of the local acetylation state. Overall, these results further support the growing body of evidence showing the importance of epigenetic modifications in the BER pathway^70,74^.

The acetyltransferase CBP/p300 acetylates both H3K18 and H3K27 *in vitro* and in cells. Thus, our results suggest that acetylation of histone H3 may represent a novel regulatory role for CBP/p300 in the BER pathway. However, given the opposing influence of H3K18ac and H3K27ac on the removal of dU from compact chromatin, the precise role of CBP/p300 in BER (i.e. suppression vs. promotion) may ultimately depend on the presence of other factors that can target its acetyltransferase activity to a specific site. For example, pre-existing histone modifications (e.g. trimethylation of H3K27) may influence the substrate specificity of CBP/p300, possibly by blocking acetylation of a specific site or altering its binding affinity to the nucleosome^75^. In addition, interactions between CBP/p300 and its numerous binding partners, including BER proteins, may promote acetylation at a particular lysine on histones. For example, thymine DNA glycosylase (TDG) was shown to significantly alter the specificity of p300, increasing its preference for H3K18^76^. It is important to note that CBP/p300 itself (which was not included in our experiments) could potentially alter DNA accessibility independent of acetylation through binding of the H3/H4 tail domains^65^. Considering the observation reported herein, as well as the intimate association of CBP/p300 with several BER factors and chromatin, there remains a compelling need for future investigations on the role of CBP/p300 in the BER pathway.

Overall, this work bring us one step closer to understanding how histone acetylation influences BER and highlights the advantages of using chemically defined nucleosome arrays to investigate the BER pathway. In the future, it will be interesting to use this model system to examine how acetylation at other histone sites (and combinations thereof), as well as other histone PTMs, influence the BER pathway. Given that BER must occur within a variety of chromatin states (both structural and chemical) throughout the genome, these studies will provide a critical first step in unraveling the relationship between histone PTMs, chromatin structure, and BER *in vivo*.

## Methods

### Preparation of DNA substrates containing dU49

Assembly of a plasmid DNA (**pUC-12-601-Nt**) containing the unmodified 12 × 601 DNA template (**12-601-Nb**) was described previously^19^. A short oligonucleotide containing a single dU residue was incorporated into the plasmid using our previously reported strand-exchange protocol^19^. Briefly, plasmid **pUC-12-601-Nt** (500 μg, 160 pmol) was digested for 1 hour with 500 units of Nb.BbvCI (all RE were purchased from New England Biolabs) in a 700 μL reaction mixture according to the manufacturer’s recommended protocol using NEB buffer 3.1 (100 mM NaCl, 50 mM Tris-HCl, 10 mM MgCl_2_, 100 μg/mL BSA, pH 7.9). To the nicked plasmid was added 3.2 nmol of the 5′-[^32^P]-labeled synthetic oligonucleotide insert (N5_dU49; Table S1) containing dU49 and 800 pmol of a locked nucleic acid (LNA) capture probe (N5_Cap.Nb; Table S1), which was required to sequester the unmodified strand. The reaction mixture was then heated at 80 °C for 20 minutes before being slowly cooled to room temperature. Following this annealing step (~1 hour), 400 units of T4 DNA ligase (New England Biolabs) and ATP (2 mM final concentration) were added to the mixture. The ligation reaction was allowed to proceed for 2 hours at room temperature, and inactivated at 70 °C for 20 minutes. Finally, the reaction mixture was desalted by ethanol precipitation and complete incorporation of the dU49-containing oligonucleotide was confirmed by 10% native PAGE (19:1 acrylamide:bisacrylamide) (Fig. S1b).

The unmodified DNA template (**1-601-Nb**) used to assemble mononucleosomes containing dU49 was prepared by PCR from the native 601 DNA sequence using primers N5_NCP_FWD and N5_NCP_REV (Fig. S2a and Table S1). The dU residue was then installed into this DNA using the same oligonucleotide exchange process described above, employing oligonucleotide N5_dU49 (Table S1). Complete incorporation of the modified oligonucleotide was confirmed by native PAGE (Fig. S2b).

### Histone preparation and octamer refolding

Recombinant human histones (H2A.1, H2B.1, H3, and H4) were expressed and purified using established protocols^77^. Homogeniously acetylated histones (H3K18ac and H3K27ac) were expressed and purified from ΔCobB *E.coli* cells (BL21[DE3] strain) as descibed previously^45^, and acetylation was confired by mass spectrometry (Fig. S3). Histone octomers were refolded using standard protocol^42–44^, and purified by size exclusion chromatography using a SuperDex 200 10/300 GL column (GE Lifesciences). Purified histone octamers were stored at 4 °C in Octamer Buffer (2 M NaCl, 5 mM BME, 0.2 mM PMSF, 1 mM EDTA, 10 mM HEPES, pH 7.8).

### Reconstitution of mononucleosomes and nucleosome arrays

Reconstitution of both mononucleosomes and nucleosome arrays was carried out according to procedures in our previous report^19^. Prior to reconstitution, the dU containing 12 × 601 DNA template (**12-601-dU49**) was removed from the corresponding plasmid backbone via digestion with EcoRV. The reaction mixture was also digested with DraI and HaeII (600 units each) in order to degrade the plasmid DNA to fragments ≤700 bp. Following digestion, the reaction mixture was passed through a QIAquick spin column (Qiagen) and the eluted DNA was used directly to reconstitute nucleosome arrays. Following the reconstitution step, oligonucleosomes were purified away from the plasmid DNA using selective Mg^2+^ precipitation (6 mM MgCl_2_). After addition of MgCl_2_, the samples were incubated on ice for 30 minutes and spun at 13,000 × g for 20 minutes at 4 °C. The pellet containing the pure arrays was then dissolved in 25 µL of low salt (LS) buffer (25 mM NaCl, 0.1 mM PMSF, 10 mM HEPES, pH 7.8) and stored at 4 °C until use. Reconstituted arrays were analyzed by 0.6% agarose gel electrophoresis (Fig. S4a) and reconstituted mononucleosomes were analyzed by 5% native PAGE (59:1 acrylamide:bisacrylamide) (Fig. S5).

### Nucleosome saturation assay

Nucleosome saturation was confirmed by digestion of 0.2 pmol oligonucleosome array with 10 units of PflMI/BstXI in LS buffer supplemented with 2 mM MgCl_2_. The respective naked DNAs were also digested under the same conditions, and both sets of samples (naked DNA and arrays) were analyzed side-by-side by 5% native PAGE (59:1 acrylamide:bisacrylamide) (Fig. S4b). The presence of a nucleosome band as well as the absence of significant free DNA (<3%) demonstrates full nucleosome occupancies in these reconstituted arrays.

### Partial micrococcal nuclease digestion of reconstituted arrays

The presence of 12 nucleosomes per array was confirmed through partial micrococcal nuclease (MNase) digestion. Arrays (0.4 pmol) were digested with 1000 units of MNase for 1 min on ice in presence of 5 mM CaCl_2._ Reactions were stopped with the addition of 0.2% (v/v) SDS and 20 mM EDTA and filtered through a QIAquick spin column. The eluted DNA was analyzed on a 0.5% agarose gel and visualized with ethidium bromide (Fig. 2b).

### UDG and APE1 digestion reactions

Reaction mixtures contained 10 nM of the appropriate substrate, 25 mM NaCl, 0.1 mM PMSF, 10 mM HEPES, pH 7.8, and either 0.2 or 2 mM MgCl_2_. Reactions were initiated by adding UDG and APE1 (1 nM each, New England Biolabs) and incubated at 37 °C for the indicated times. For mononucleosomes, reactions were supplemented with 110 nM (11 equivalents) of undamaged mononucleosomes such that the ratio of damaged to undamaged mononucleosomes, as well as total number of 601 units, was identical to the 12 × 601 arrays. Aliquots were removed at different times and the reaction stopped with the addition of 1% SDS (v/v), NaBH_4_ (10 mM), and 5 units proteinase K. The mixture was then extracted with phenol:chloroform:isoamyl alcohol (25:24:1 v/v, ThermoFisher Scientific) and the DNA desalted by ethanol precipitation. The resulting DNA was digested with 5 units of Nb.BbvCI in order to excise the dU containing 5′-[^32^P]-labeled oligonucleotide insert from the large 12 × 601 DNA array. The digested DNA was then resolved by 10% denaturing PAGE (19:1 acrylamide:bisacrylamide) and visualized using a Typhoon FLA 9500 gel imager (GE Healthcare) (Fig. S6). The fraction cleaved was quantified using ImageQuant TL software (GE Healthcare, *v*8.1). At least three replicates were carried out for each substrate, and initial rates were calculated by fitting the digestion data in to a single phase exponential and multiplying each rate constant by the *Y*_max_ as previously described^12,63^.

### Analysis of chromatin compaction by FRET

We prepared a fluorescently labeled 12 × 601 DNA template by installing Cy3 (Donor) and Cy5 (Acceptor) dyes within N7 and N5, respectively, as described in our previous report^19^. This DNA (**12-601-FRET**) was reconstituted using either WT histone octamers or octamers containing acetylated histone H3 (Fig. S7). FRET measurements on the resulting chromatin were carried out as previously described^68^. Briefly, nucleosome arrays (10 nM) were equilibrated in LS buffer in the presence (2 mM) or absence of MgCl_2_ at 37 °C for 5 minutes. A portion of each solution (20 μL) was then transferred to a Nunc 384-Well Optical Bottom Plate (Thermofisher) for imaging. Plates were siliconized with Sigmacote (Sigma Aldrich) according to the manufacturer’s instructions prior to use. Samples were imaged with a Typhoon FLA 9500 multimode imager (GE Healthcare Lifesciences) and all FRET intensities were corrected for spectral overlap as described previously^68,78^.

### Restriction enzyme accessibility assay

The indicated nucleosome arrays (5 nM) were digested with either BstXI (0.1 U/μL) or BbvCI (0.2 U/μL) in LS buffer supplemented with 2 mM MgCl_2_ at 37 °C. Aliquots were taken at the indicated times and quenched with three volume equivalence of LS buffer supplemented with 0.1% SDS, 2 u/μL Proteinase K, and 8% glycerol and then were heated at 60 °C for 10 minutes. Digested DNA was resolved by 1% agarose gel electrophoresis and visualized/quantified as described above (Fig. S8).

### Statistical analysis

All statistical analysis was conducted using GraphPad Prism (v7.03). Corrected FRET intensities were compared by an unpaired one-way analysis of variance (ANOVA). Rates and yields of UDG/APE1, BstXI, and BbvCI cleavage reactions at each Mg^2+^ concentration (0.2 mM and 2 mM) were compared using an unpaired one-way ANOVA. Prior to the analysis, each data set was separately screened for outliers using a Grubbs test (α = 0.01). Within individual Mg^2+^ concentrations, rates and product yields on mononucleosomes and arrays were then compared to free DNA using a Dunnett’s multiple comparisons test. Acetylated and non-acetylated substrates were compared similarly, as were arrays and corresponding mononucleosomes. P-values less than 0.05 were considered significant.

## Supplementary information

Supplementary Information

## Data Availability

The data generated or analyzed during the current study are available from the corresponding author on reasonable request.

## Supplementary information

is available at this paper at 10.1038/s41598-019-52340-0.
