# Supplementary material for: Acetylation of the histone H3 tail domain regulates base excision repair on higher-order chromatin structures
Source: Sci Rep. 2019 Nov 4;9:15972. doi: 10.1038/s41598-019-52340-0 (PMC6828659; doi:10.1038/s41598-019-52340-0)
Supplement: Supplementary file 1 — Supplementary Information [file 41598_2019_52340_MOESM1_ESM.pdf]

Supplementary Information

Acetylation of the histone H3 tail domain regulates base excision repair on higher-order chromatin structures

Deb Ranjan Banerjee[2†], Charles E. Deckard III[1†], Yu Zeng[3], and Jonathan T. Sczepanski[1,*]

[1]Department of Chemistry, Texas A&M University, College Station, Texas 77843, United States

[2]Current address: Department of Chemistry, National Institute of Technology, Durgapur, West Bengal, India

[3]Current address: Department of Microbial Pathogenesis and Immunology, Texas A&M University, College Station, Texas, 77843, United States

†These authors contributed equally.

S1. Supplementary Figures

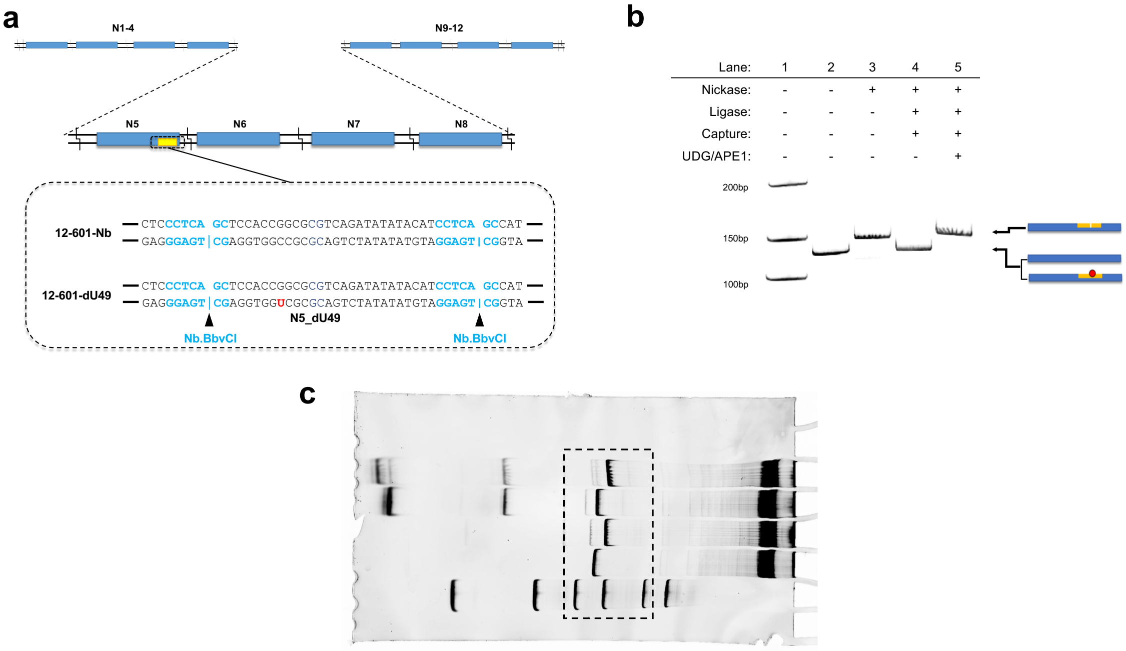

Figure S1. Sequence details for the 12×601 DNA template **12-601-dU49**. (a) Schematic representation of the position of dU lesions within **12-601-dU49**. **12-601-dU49** was constructed from **12-601-Nb** via strand exchange with N5_dU49 (see Methods and Table S1). Light blue sequence represents the Nb.BbvCI recognition sites. Sequence details for the entire 12×601 DNA construct can be found in our previous report.[1] (b) Representative electrophoretic mobility shift assay demonstrating the insertion of a dU containing oligonucleotide N5_dU49 into template **12-601-Nb** (i.e. **12-601-dU49**). All reactions were carried out on the corresponding plasmid DNA as described previously[1] and the 601 DNA fragment (N5) containing the modified site was excised via PflMI and BstXI restriction digestion prior to analysis by 10% native PAGE (19:1 acrylamide:bisacrylamide). Lane 1, DNA ladder; lane 2, unmodified **12-601-Nb**; lane 3, **12-601-Nb** DNA treated with Nb.BbvCI; lane 4, ligated **12-601-dU49** following strand exchange with N5_dU49; lane 5, UDG/APE1 treatment of ligated material from lane 4. (c) Uncropped gel image for Figure S1b. The box indicates the cropped region shown in Figure S1b.

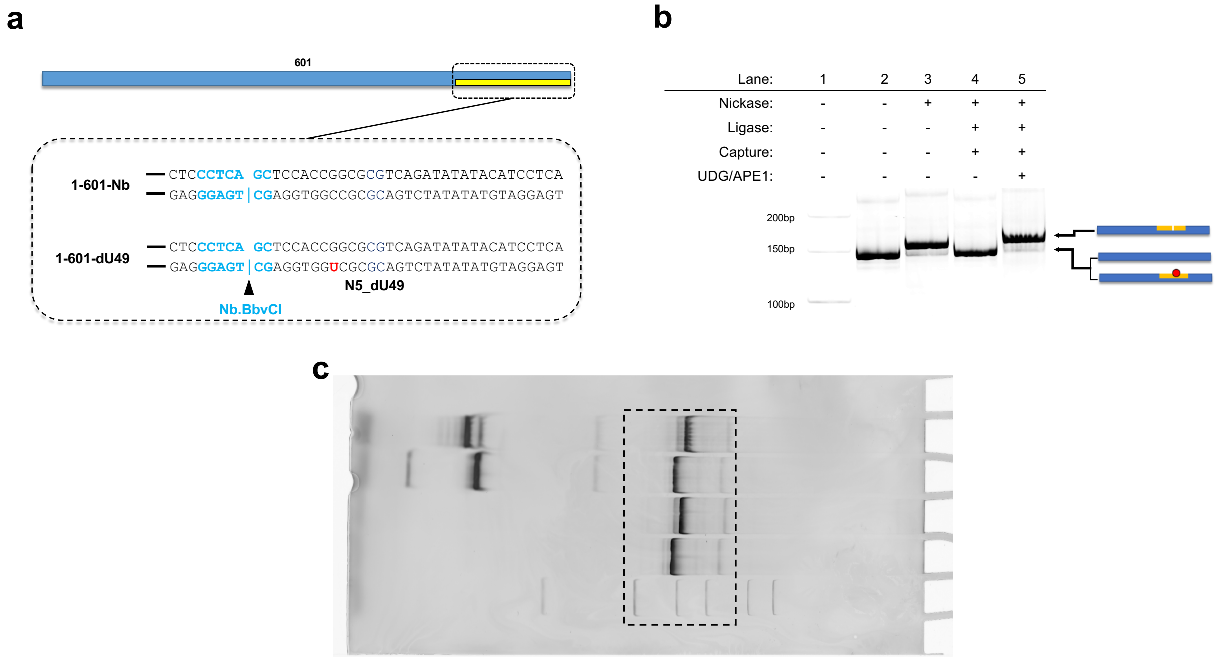

Figure S2. Sequence details for the mononucleosome DNA template **1-601-dU49**. (a) Schematic representation of the unmodified 601 DNA template (**1-601-Nb**) used to prepare mononucleosome **1-NCP-dU49,** which is identical to nucleosome 5 (N5) within array **12-NCP-dU49**. **1-601-dU49** was constructed from **1-601-Nb** via strand exchange with N5_dU49 (see Methods and Table S1). Light blue sequence represents the Nb.BbvCI recognition sites. (b) Representative electrophoretic mobility shift assay demonstrating the insertion of a dU containing oligonucleotide N5_dU49 into template **1-601-Nb** (i.e. **1-601-dU49**). All reactions were carried out as described previously[1] and the DNA was analyzed by 10% native PAGE (19:1 acrylamide:bisacrylamide). Lane 1, DNA ladder; lane 2, unmodified **1-601-Nb**; lane 3, **1-601-Nb** DNA treated with Nb.BbvCI; lane 4, ligated **1-601-dU49** following strand exchange with N5_dU49; lane 5, UDG/APE1 treatment of ligated material from lane 4. (c) Uncropped gel image for Figure S2b. The box indicates the cropped region shown in Figure S2b.

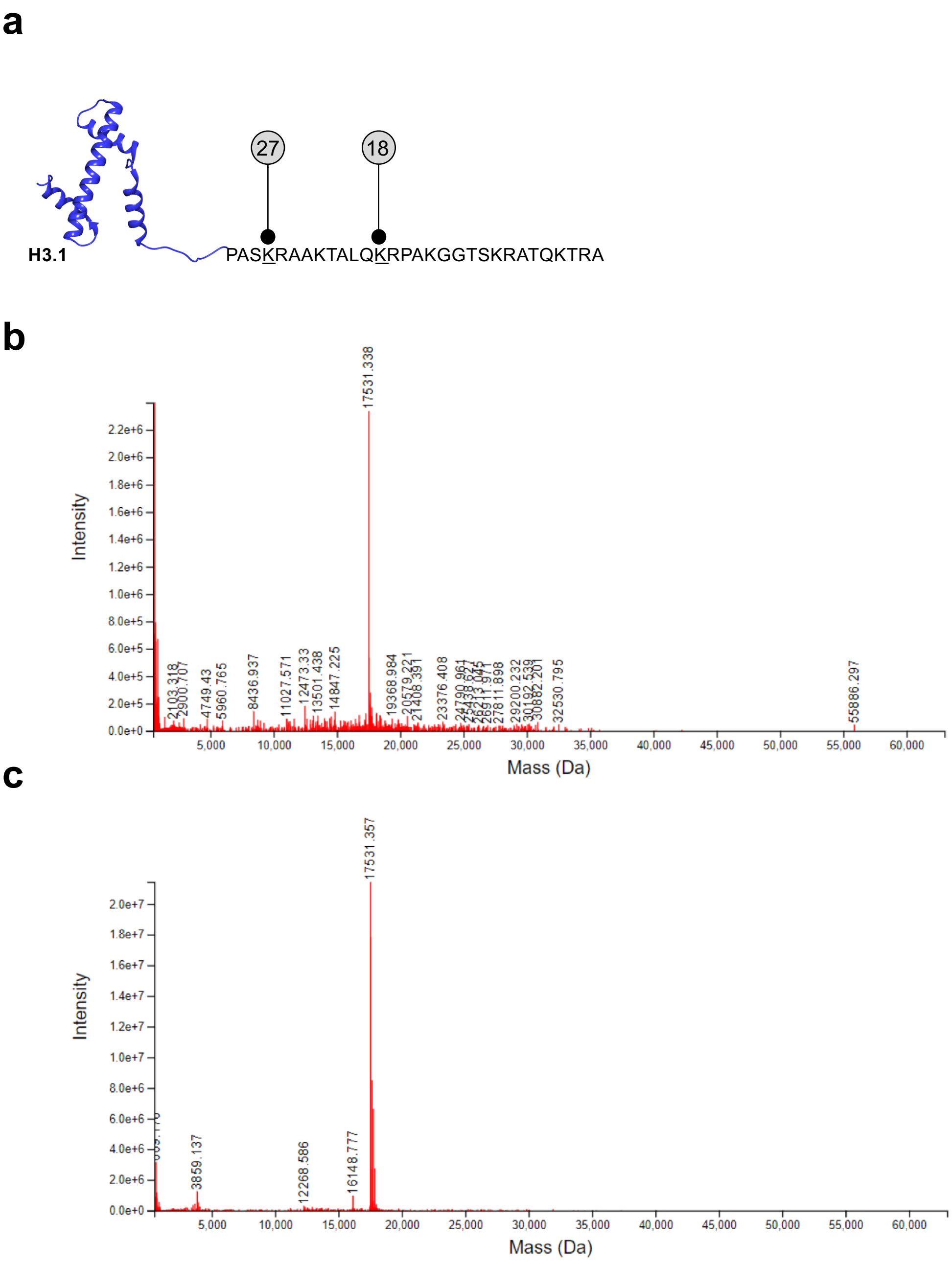

Figure S3. Characterization of homogeneously acetylated histone H3. (a) Acetylation sites on the histone H3 tail domain studied in this work (PDB: 1kx5). (b) ESI-MS spectra of histone H3 homogeneously acetylated at lysine 18 (H3K18ac). Calculated: 17532.473 Da; Measured: 17531.338 Da. (c) ESI-MS spectra of histone H3 homogeneously acetylated at lysine 27 (H3K27ac). Calculated: 17532.473 Da; Measured: 17531.357 Da.

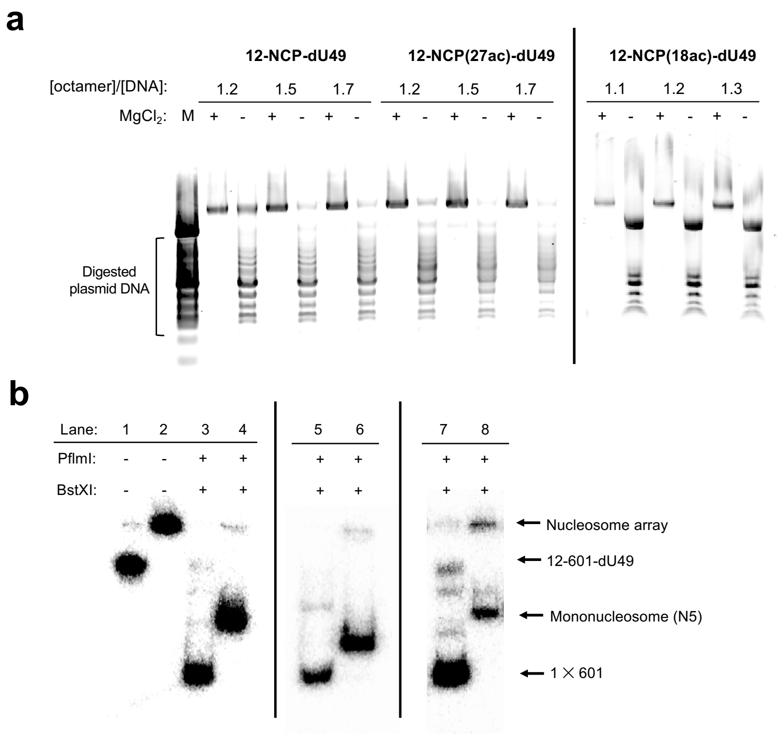

Figure S4. Characterization of reconstituted nucleosome arrays. (a) Agarose gel electrophoresis (0.6% in 0.2× TBE) analysis of the 12×601 DNA template (**12-601-dU49**) reconstituted with increasing concentrations of WT or homogeneously acetylated (H3K18ac and H3K27ac) histone octamers. The molar ratio of histone octamer to DNA template is indicated. For each ratio, the reconstitution reaction was analyzed before (-) and after (+) purification by Mg^{2+} induced precipitation ([Mg^{2+}]). M: digested (EcoRV) plasmid DNA (**pUC-12-601-Nt**) showing the release of **12-601-dU49** DNA tamplate. (b) Restriction digestion analysis (5% native PAGE, 59:1 acrylamide:bisacrylamide) of reconstituted nucleosome arrays. Nucleosome arrays **12-NCP-dU49** (lane 4), **12-NCP(27ac)-dU49** (lane 6), and **12-NCP(18ac)-dU49** (lane 8) were digested with PflMI/BstXI. **12-601-dU49** DNA (lanes 3, 5, and 7) was digested similarly and run side-by-side with the digested arrays. Undigested **12-601-dU49** (lane 1) and **12-NCP-dU49** (lane 2) were used as controls. Vertical black lines indicate images taken from different gels. Uncropped gel images are shown in Figure S10.

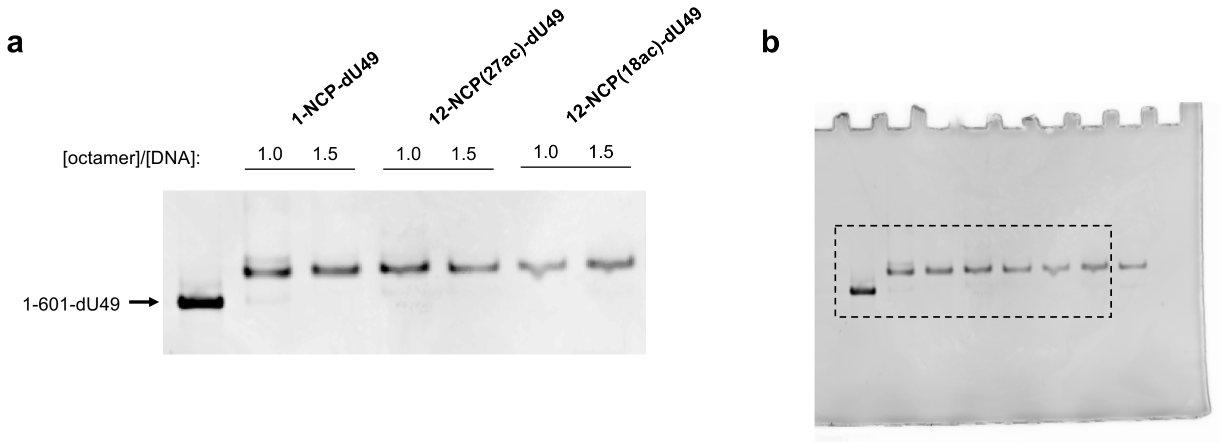

Figure S5. Reconstitution of mononcleosomes. (a) Agarose gel electrophoresis (0.6% in 0.2× TBE) analysis of the 1×601 DNA template (**1-601-dU49**) reconstituted with increasing concentrations of WT or homogeneously acetylated (H3K18ac and H3K27ac) histone octamers. The molar ratio of histone octamer to DNA template is indicated. (b) Uncropped gel image for Figure S5a. The box indicates the cropped region shown in Figure S5a.

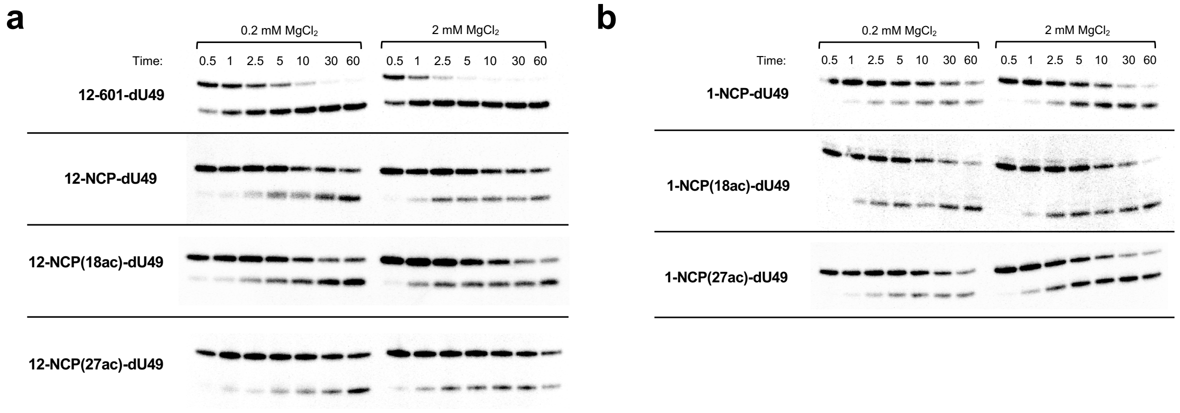

Figure S6. Representative gels (10 % denaturing PAGE, 19:1 acrylamide:bisacrylamide) of nucleosome arrays (a) and mononucleosomes (b) following treatment with UDG/APE1. Reactions were carried out as described in the Methods section of the main text. Horizontal black lines indicate images taken from different gels. Uncropped gel images are shown in Figure S11.

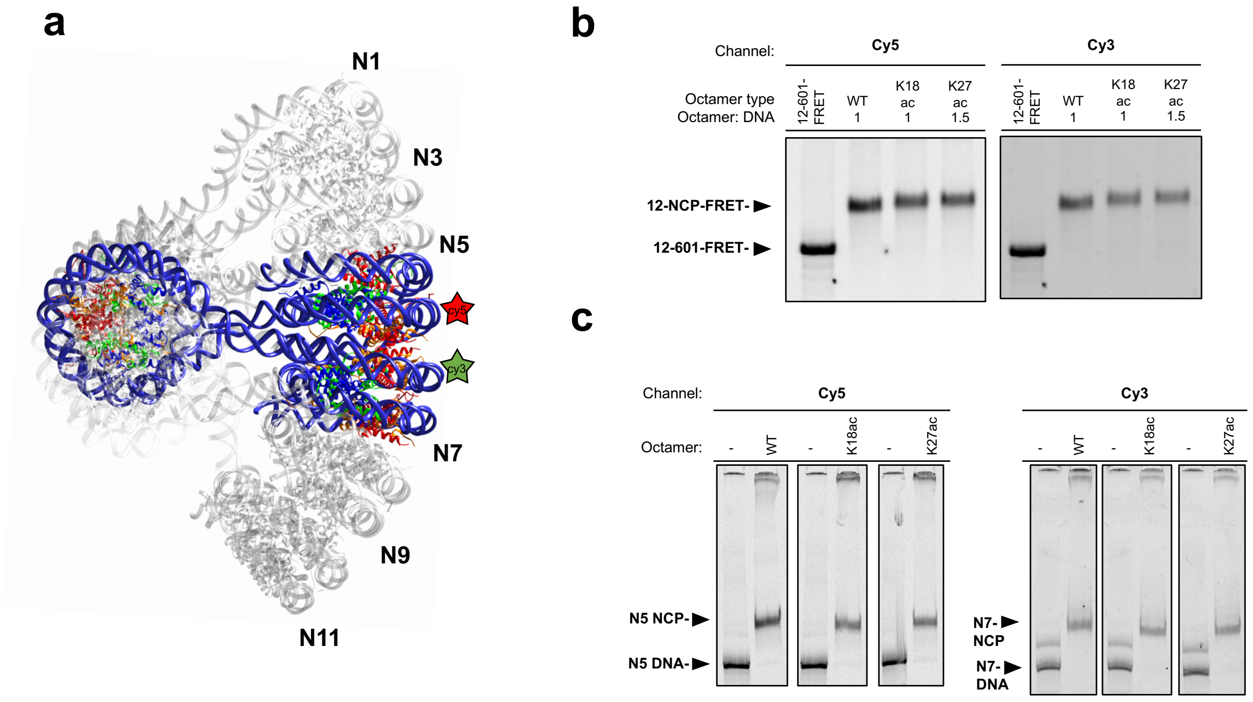

Figure S7. Characterization of Cy3/Cy5-labelled chromatin. (a) Structural representation of the dye-labeled arrays used in FRET experiments; the DNA (**12-601-FRET**) used to assemble these arrays was reported previously.[1,2] (b) Agarose gel electrophoresis (0.6%) analysis of **12-601-FRET** reconstituted with the indicated ratio of histone octamer to DNA template. Images from the Cy3 channel (532 nm ex.; 575 nm em.; right) and Cy5 channel (635 nm ex.; 665 nm em.; left) are shown. (c) Restriction digestion analysis of nucleosome occupancy of **12-NCP-FRET** reconstituted with the indicated histone octamer (5% native PAGE, 59:1 acrylamide:bisacrylamide). Both naked DNA (**12-601-FRET**) and the indicated arrays were digested with either PflMI/BstXI (to remove N5) or DraIII/BstEII (to remove N7) and were then run side-by-side to determine nucleosome occupancy at these sites. WT: canonical octamer; K18ac: octamer containing histone H3 acetylated at lysine 18; K27ac: octamer containing histone H3 at lysine K27. Uncropped gel images are shown in Figure S12.

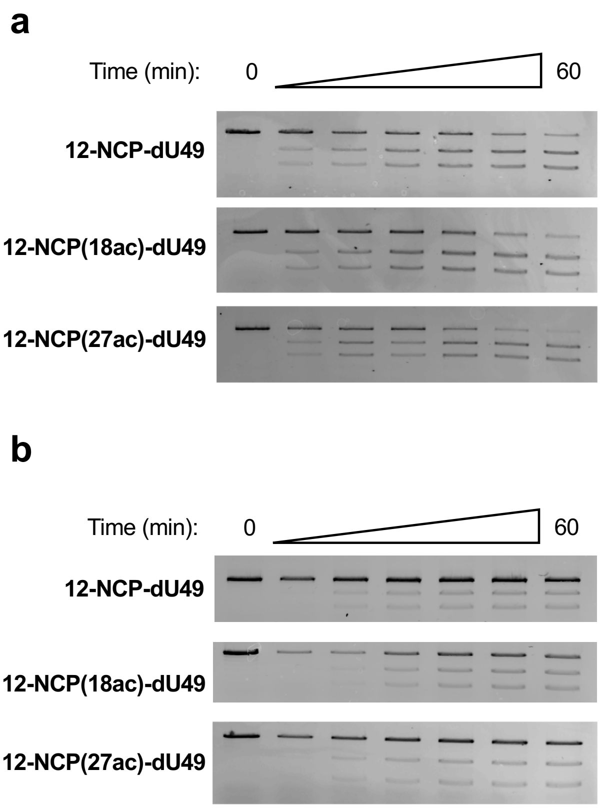

Figure S8. Restriction enzyme accessibility assays on WT and homogeneously acetylated nucleosome arrays. (a,b) Representative gel images showing digestion of the indicated substrates by either BstXI (a) or BbvCI (b). Uncropped gel images are shown in Figure S13.

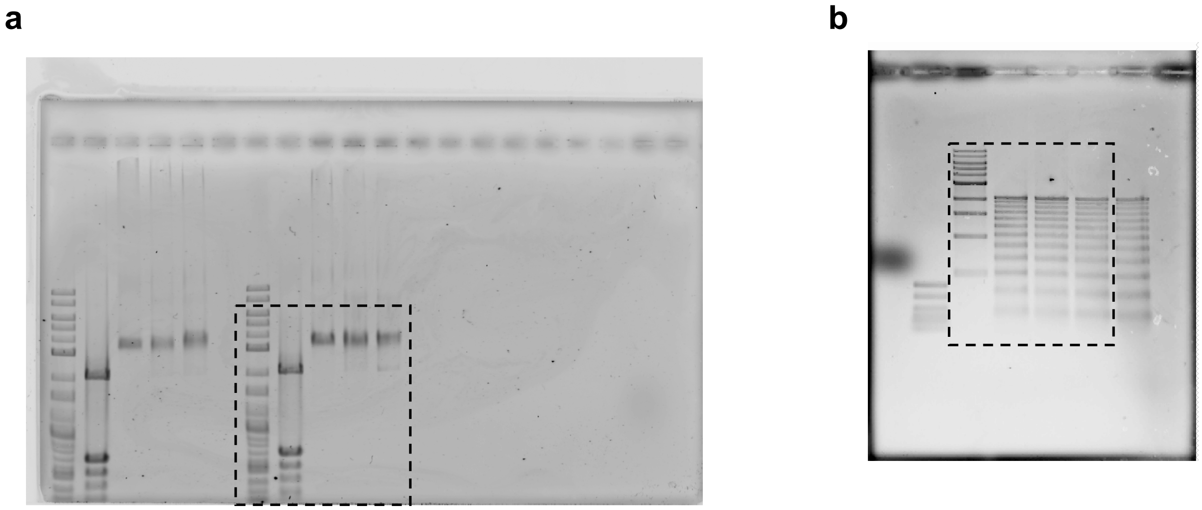

Figure S9. Uncropped gel images for Figure 2a (a) and Figure 2b (b) in the main text. Boxes indicate the cropped regions shown in Figure 2.

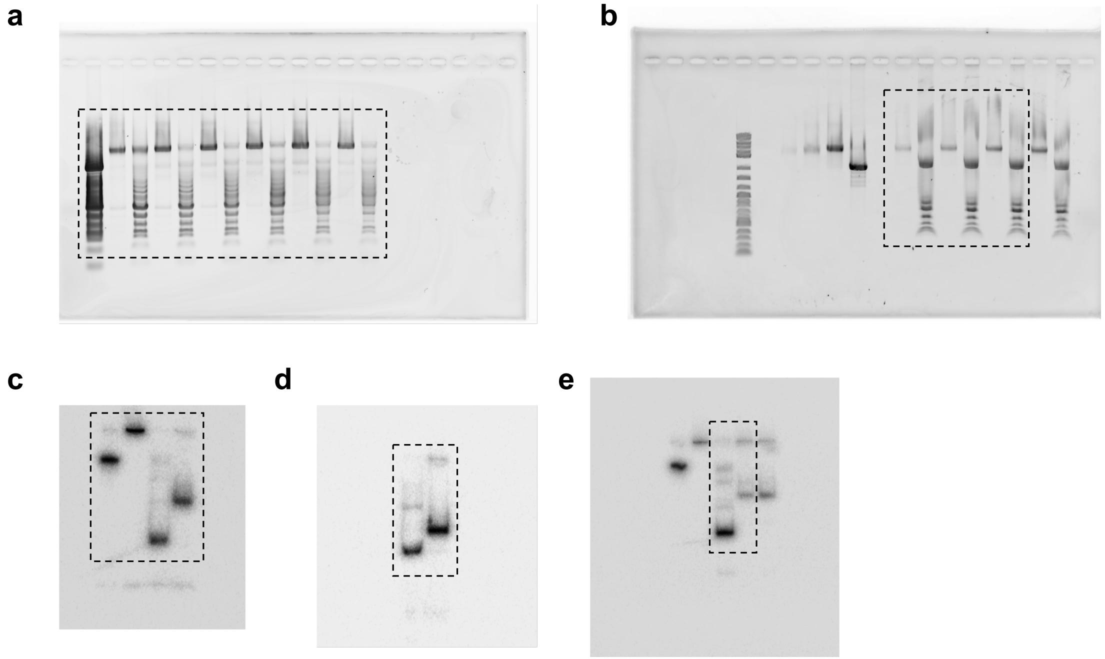

Figure S10. Uncropped gel images for Figure S4. (a) and (b) are uncropped gel images for Figure S4a (left) and Figure S4a (right), respectively. (c), (d) and (e) are uncropped gel images for Figure S4b (left), Figure S4b (middle), and Figure S4a (right), respectively. Boxes indicate the cropped regions shown in Figure S4.

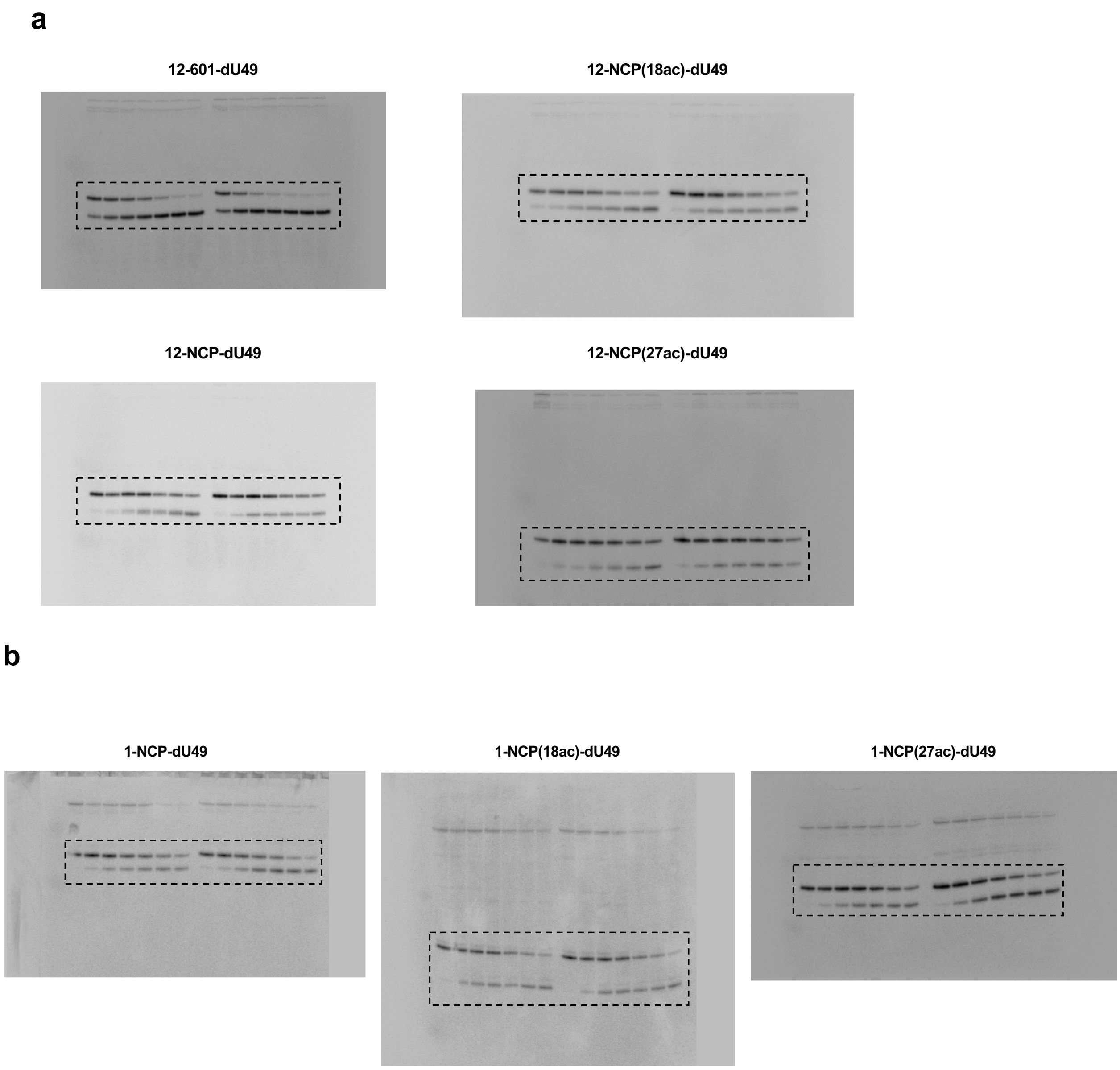

Figure S11. Uncropped gel images for Figure S6a (a) and Figure S6b (b). Boxes indicate the cropped regions shown in Figure S6.

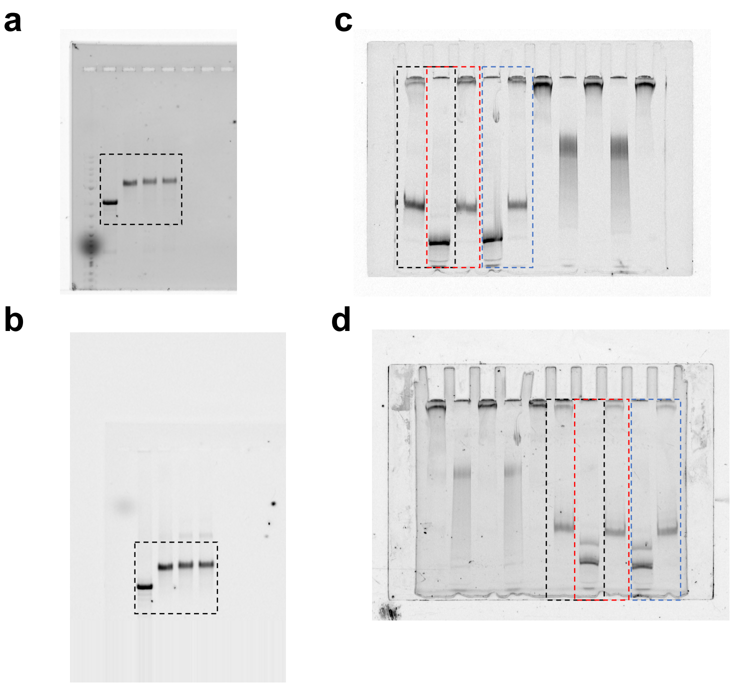

Figure S12. Uncropped gel images for Figure S7b (a,b) and Figure S7c (c,d). Gels were scanned using the Cy3 channel (532 nm ex.; 575 nm em.; a, c) and Cy5 channel (635 nm ex.; 665 nm em.; b, d). Boxes indicate the cropped regions shown in Figure S7. For panels c and d, black, red, and blue boxes indicate panels WT, H3K18, and H3K27 in Figure S7, respectively.

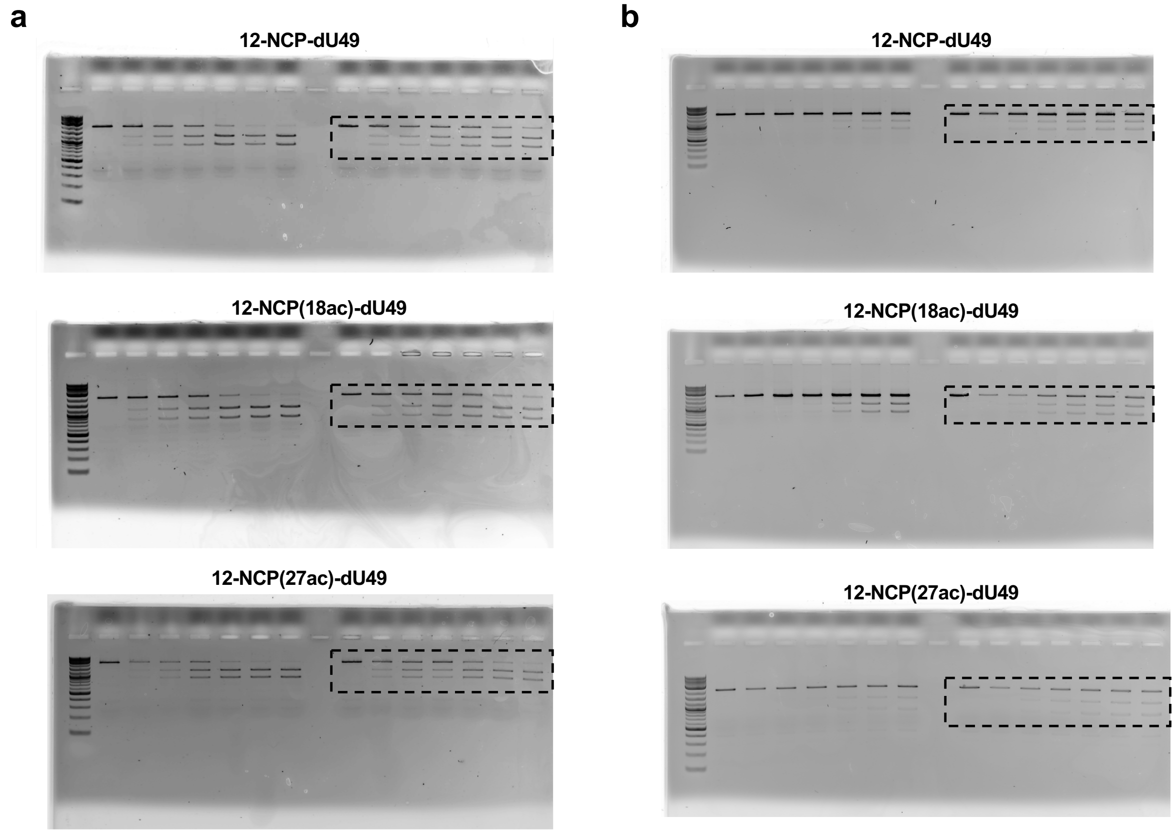

Figure S13. Uncropped gel images for Figure S8a (a) and Figure S8b (b). Boxes indicate the cropped regions shown in Figure S8.

S2. Supplementary Table.

Table S1. Sequences of oligonucleotides described in this study. The underlined nucleotides correspond to locked nucleic acids (LNA).

Name	Sequence (5′ to 3′)
N5_dU49	pTGAGGATGTATATATCTGACGCGC/dU/GGTGGAGC
N5_Cap.Nb	GCTCCACCGGCGCGTCAGATATATACATCCTCA
N5_NCP_Fwd	CTGGAGAATCCCGGTGCCGAGGCC
N5_NCP_Rev	TGAGGATGTATATATCTGACGCGCCGGTGGAGC

S3. References

(1) Banerjee, D. R. *et al.* Plug-and-Play Approach for Preparing Chromatin Containing Site-Specific DNA Modifications: The Influence of Chromatin Structure on Base Excision Repair. *J. Am. Chem. Soc.* **140**, 8260–8267 (2018).

(2) Deckard, C. E., Banerjee, D. R. & Sczepanski, J. T. Chromatin structure and the pioneering transcription factor FOXA1 regulate TDG-mediated removal of 5-formylcytosine from DNA. *J. Am. Chem. Soc.* **141**, 14110–14114 (2019).
